# Treatment of textile wastewater using the Co(II)/NaHCO_3_/H_2_O_2_ oxidation system

**DOI:** 10.1016/j.heliyon.2023.e22444

**Published:** 2023-11-17

**Authors:** Francisco J. Ariza-Pineda, Iván F. Macías-Quiroga, Diego F. Hinojosa-Zambrano, Juan D. Rivera-Giraldo, Diana M. Ocampo-Serna, Nancy R. Sanabria-González

**Affiliations:** a Departamento de Ingeniería Química, Universidad Nacional de Colombia Sede Manizales, Campus La Nubia, km 7 vía al Aeropuerto, Manizales, Colombia; b Departamento de Física y Química, Universidad Nacional de Colombia Sede Manizales, Campus La Nubia, km 7 vía al Aeropuerto, Manizales, Colombia; c Departamento de Química, Facultad de Ciencias Exactas y Naturales, Universidad de Caldas, Calle 65 N° 26-10, AA 275, Manizales, Colombia

**Keywords:** Textile wastewater, Acid Black 194, Bicarbonate-activated peroxide, Cobalt, Degradation, Toxicity

## Abstract

Textile wastewater (TWW) is one of the most hazardous wastewaters for ecosystems when it is discharged directly into water streams without adequate treatment. Some organic pollutants, such as dyes in TWW, are considered refractory compounds that are difficult to degrade using conventional chemical and biological methods. The bicarbonate-activated peroxide (BAP) system is an advanced oxidation process (AOP) based on applying H_2_O_2_, which has been demonstrated to be a clean and efficient technology for dye degradation, with the advantage of operating under slightly alkaline pH conditions. In this study, response surface methodology (RSM) based on a central composite design (CCD) was used to optimize the degradation of TWW contaminated with the azo dye Acid Black 194 using the BAP system catalyzed with cobalt ions in solution (Co^2+^). The analysis of variance (ANOVA) technique was applied to identify significant variables and their individual and interactive effects on the degradation of TWW. The optimum reagent concentrations for degrading TWW at 25 °C and with 45 μM Co^2+^ were 787.61 and 183.34 mM for H_2_O_2_ and NaHCO_3_, respectively. Under these conditions, complete decolorization (≥99.40), 32.20 % mineralization, and 52.02 % chemical oxygen demand removal were achieved. Additionally, the acute toxicity of textile wastewater before and after oxidation was evaluated with guppy fish (*Poecilia reticulata*), showing a total reduction in mortality after treatment with the Co^2+^–BAP system. The Co^2+^–BAP oxidation system is a potential method for textile wastewater treatment, which, in addition to achieving complete decolorization and partial mineralization, improves biodegradability and reduces the toxicity of the treated water.

## Graphical abstract

**Figure:**
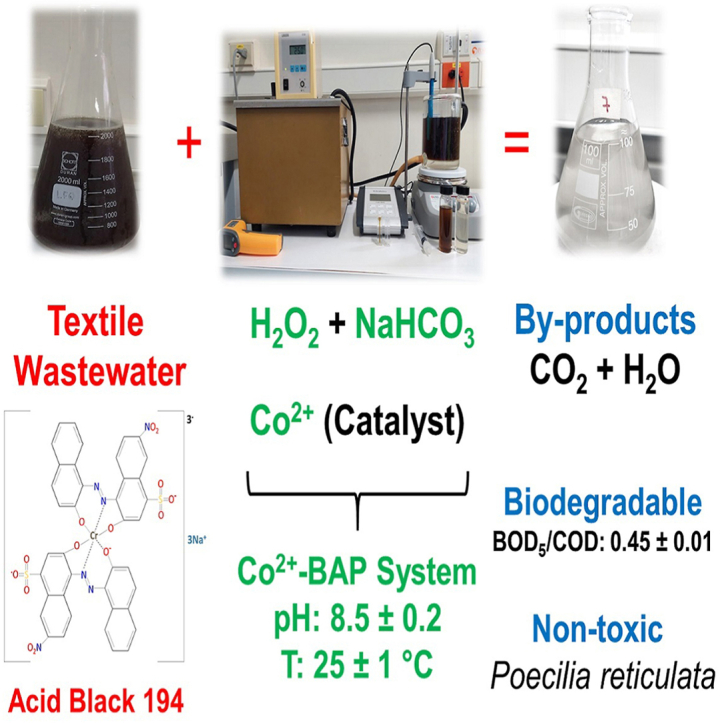

## 1 Introduction

The textile industry is essential to the development of the global economy, generating approximately one trillion dollars annually, contributing 7 % of all world exports, and employing approximately 35 million people worldwide [1,2]. However, this industry generates 54 % of the dye effluents present in the environment, followed by dyeing (21 %), pulp and paper (10 %), tanning and painting (8 %), and dye manufacturing industries (7 %) [3,4]. In 2015, the global textile sector estimated its water consumption at 79 billion cubic meters, which is expected to increase by 50 % by 2030 [5].

Wastewater generated by the textile industry includes mainly cleaning and processing water, and its characteristics depend on the specific operation processes in the factory and the equipment used. Chemical components such as alkalis, acids, bleaching, enzymes, starch, dyes, resins, solvents, waxes, and oils are used in the various stages of textile processing. Hence, most textile wastewater (TWW) has high levels of chemical oxygen demand (COD), biological oxygen demand (BOD_5_), and total dissolved solids (TDS), as well as a very intense color [6].

It is estimated that 700,000 tons of dyes are manufactured annually worldwide, and approximately 100,000 dyes are marketed [[7], [8], [9], [10]], of which the most widely used are azo dyes, accounting for 65–70 % of the total dyes produced [11]. The textile industry uses the largest amount of dyes, approximately 10,000 tons/year globally [7,12]. Synthetic dyes are classified based on their chemical structure, solubility, and application method on different substrates [3]. According to their chemical structure, dyes have been grouped into eight main categories: azo, anthraquinone, indigo, triarylmethane, heterocyclic, cyanine, sulfur, and phthalocyanine [13]. Acid, basic, direct, metalliferous, and reactive dyes are water-soluble dyes; disperse, sulfur, solvent, and vat dyes are some examples of water-insoluble dyes [3,14]. Application-based classification has been the primary system adopted by the color index (C.I.) and is advantageous due to the complexities of dye chemical nomenclature [11].

Acid dyes, especially those containing the functional group $–SO_{3}^{–}$, are widely used in the textile, pharmaceutical, printing, leather, dyeing, paper, and other sectors due to their bright colors and high solubility [15,16] and constitute 30–40 % of the total dye consumption [15]. The global acid dye market is expected to grow from $3.60 billion in 2022 to $4.01 billion in 2023 at a compound annual growth rate (CAGR) of 11.6 % [17]. Acid dyes used in the textile industry are water-soluble anionic compounds, mostly applied on natural fibers such as wool, silk, mohair, angora, and alpaca, and synthetic polyamide such as nylon, slightly applied to acrylic and blended materials [3]. The main advantages of using these dyes are that they are easy to apply, inexpensive, and have moderate fastness in dry cleaning. However, the affinity of acid dyes increases considerably when a mordant (mainly chromium salts) is applied to the fiber before applying the dye [18].

Metal-complex dyes (also called premetallized acid dyes) have a mordant metal incorporated within the dye molecule and can be of two classes: 1:1 metal complexes and 1:2 metal complexes [19,20]. The metal-complex dye contains a metal atom (usually chromium, cobalt, or copper) coordinated with one or two molecules of a dye ligand [19]. Acid Black 194 (AB–194) is a 1:2 symmetric metal-complex dye with a central chromium(III) atom coordinated with two organic ligand molecules [21]. AB–194 is one of the most popular commercial dyes for dyeing leather, wool, polyamide, and silk, among other materials [21,22]. The organic ligand is an azo dye that allows multiple coordination with the metal ion and confers to the complex multiple negative charge that ensure good solubility of the dye in water [21].

Different processes have been used for the elimination/degradation of water polluted with azo dyes [23,24], such as coagulation/flocculation [6], adsorption [25], electrochemical treatments [26], and advanced oxidation processes (AOPs) [[27], [28], [29]]. As TWW is usually characterized by a low BOD_5_/COD ratio, typically between 0.06 and 0.35 [[30], [31], [32]], and a ratio below 0.4 indicates low biodegradability [30], AOPs have been successfully used for the degradation of these wastewaters [29,33]. Among the versatile AOPs, Fenton, Fenton-like, and photo-H_2_O_2_ are considered the most efficient technologies for treating organic pollutants in wastewater [34,35]. Metal ions/oxides (Fe, Mn, Co, Cu, etc.) have been used as catalysts in H_2_O_2_-based AOP activation to generate highly oxidizing species such as hydroxyl radicals (•OH), and during this process, the •OH favors the degradation and mineralization of pollutants [[36], [37], [38]].

Although H_2_O_2_-based AOPs have demonstrated effectiveness in treating a wide variety of wastewater [36,39], their application has a limited pH range, between 2 and 4 [35,36]. The bicarbonate-activated hydrogen peroxide (BAP) system is an AOP that operates at neutral or slightly alkaline pH (pH 6–9). In comparison to H_2_O_2_-based AOPs, the BAP system generates more reactive oxygen species (ROS), including peroxymonocarbonate ion (HCO_4_^−^), superoxide ion ($O_{2}^{•-}$), singlet oxygen (^1^O_2_) and carbonate radical anion (${\text{CO}}_{3}^{•-}$) [36,40]. The BAP system is considered a clean, cost-effective, and efficient technology that could further expand the application of H_2_O_2_-based AOPs [36]. This system has shown excellent results in the treatment of colored waters [35,41], especially those catalyzed by cobalt ions (Co^2+^–BAP) [[42], [43], [44], [45], [46], [47]]. Rapid and nearly complete decolorization of methylene blue [42,45], reactive brilliant red X–3B [43], acid orange II [44,46], and Ponceau 4R [47] has been achieved using the Co^2+^–HCO_3_^–^–H_2_O_2_ system. The activity in the Co^2+^–BAP system has been attributed to the formation of active complexes between cobalt and HCO_3_^−^, which produce various ROS upon reaction with H_2_O_2_ [[42], [43], [44], [45], [46], [47]].

Most studies on dye degradation with the Co^2+^–BAP system have analyzed the effect of process variables using the conventional approach, i.e., changing one variable at a time while keeping all others at a constant value [[41], [42], [43],^45^,^46^]. This classic approach is time-consuming, requires many experiments, and cannot represent the interactive effects of the process variables [48,49]. Response surface methodology (RSM) consists of a group of mathematical and statistical tools used to model and analyze problems requiring a desirable response affected by multiple variables [50,51]. RSM is often applied to analyze the influence of various experimental parameters and examine their effects on the reaction process. It is a reliable analytical method superior to traditional methods, such as one-factor-at-a-time [50,52]. RSM has been utilized to predict the efficiency of AOPs for wastewater treatment under different operating conditions and to determine the optimal conditions that allow for the highest degradation of pollutants [[53], [54], [55], [56], [57], [58], [59]]. The central composite design (CCD), Box‒Behnken, and D-optimal designs are only three of the multiple designs used by RSM [48].

CCD and RSM were selected to study the degradation of textile wastewater with the Co^2+^-BAP oxidation system. The textile wastewater contained the pollutant Acid Black 194, a metal-complex dye, and prior to the oxidation reaction, it was treated by a coagulation/flocculation process. Although RSM-based CCD has been employed to optimize Ponceau 4R decolorization with the Co^2+^–BAP system [47], this is the first study using this AOP with a real textile wastewater sample and not a dye molecule. Optimal reagent concentrations (H_2_O_2_ and NaHCO_3_) were determined to maximize decolorization, mineralization, and chemical oxygen demand (COD) removal. Additionally, acute fish toxicity tests (*Poecilia reticulate*) were performed in textile wastewater before and after oxidation. Gas chromatography coupled to mass spectrometry (GC‒MS) analysis was used to identify some byproducts of the oxidation reaction.

## 2 Materials and methods

### 2.1 Reagents

Acid Black 194 was supplied by the local textile industry and acquired from Emperor Chemical Co., Ltd. (China). The chemical structure and properties of this dye are shown in Table 1. Hydrogen peroxide (H_2_O_2_, 30 %), sodium bicarbonate (NaHCO_3_, ≥99.7 %) and cobalt chloride hexahydrate (ClCo_2_·6H_2_O, ≥98 %) were of analytical grade and obtained from Merck KGaA (Germany). Helium (99.996 %) was used as a carrier gas in the GC‒MS analysis and was supplied by The Linde Group distributors in Colombia.

**Table 1:** Chemical structure and properties of Acid Black 194.

CAS Registry	61931-02-0	
Molecular formula	C_40_H_20_CrN_6_Na_3_O_14_S_2_	
Molecular weight	993.71 g/mol	
Appearance	Black powder	
*λ*_max_	572 nm	

The clay utilized as an adsorbent material was Colombian bentonite, which was previously characterized [60]. According to Stokes' law, the clay fraction (≤2 μm) was obtained by sedimentation and then exchanged twice with 1.0 M NaCl (99.5 %, PanReac AppliChem, Spain). This material has been shown to be efficient in the removal of Cr(III) and cobalt ions [[60], [61], [62]].

### 2.2 Real sample of textile wastewater

The raw textile wastewater corresponded to a representative sample of an industrial effluent with a high content of Acid Black 194 azo dye from the dyeing stage of a textile plant located in the Colombian coffee region (Colombia, South America). An integrated sample was collected directly from an equalization tank and stored at 4 °C prior to use. In the laboratory, textile wastewater was subjected to a coagulation/flocculation (C/F) process using aluminum sulfate tetradecahydrate (Al_2_(SO_4_)_3_.14H_2_O) and slaked lime (Ca(OH)_2_). The physicochemical characteristics of the sample were determined by following the Standard Methods (SM) for the Examination of Water and Wastewater [63], and the results obtained were analyzed using a two-tailed *t-*test (Table 2). The *p-*values obtained (with a α/2 = 0.025) in the statistical analysis were estimated using a free add-in called Real-Statistics interfaced with Microsoft Excel 365 (Microsoft, Redmond, Washington, USA).

**Table 2:** Physicochemical characteristics of textile wastewater.

Parameter	Standard method	Raw textile wastewater	After C/F	*p*-value
pH	4500-H^+^ B	8.2 ± 0.9	8.9 ± 0.3	0.0553^a^
Conductivity (μS/cm)	2510-B	2761 ± 586	4830 ± 47	0.0013^b^
Sulfates (mg/L)	4500-SO_3_^2-^-E	1310 ± 498	2679 ± 52	0.0025^b^
COD (mg O_2_/L)	5220-D	3271 ± 521	1428 ± 84	0.0016^b^
BOD_5_ (mg O_2_/L)	5210-B	648 ± 141	419 ± 32	0.0186^b^
BOD_5_/COD		0.20 ± 0.01	0.29 ± 0.01	–
TOC, mg C/L	5310-B	1271 ± 253	1083 ± 68	0.1807^a^
TSS, mg/L	2540-D	55.6 ± 24.2	12.4 ± 1.6	0.0169^b^
Acid Black 194, mg/L		928 ± 161	16.6 ± 1.2	0.0005^b^
Total chromium, mg/L	3111-B	25.9 ± 9.7	0.9 ± 0.1	0.0055^b^
Apparent color, U. Pt–Co	2120-B	45850 ± 6182	2545 ± 21	0.0002^b^

a Non-significant difference.

b Significant difference.

### 2.3 Experimental design - oxidation tests

Response surface methodology is a set of statistical and mathematical methods to optimize processes. Using the RSM, it is possible to design experiments, create models, estimate the effect of different factors (variables), and finally identify the optimal operating conditions using the least number of experiments [57,64]. One of the most widely used RSM methods is central composite design (CCD) [53,[55], [56], [57],^65^,^66^]. This two-level factorial method includes central and axial points, making it possible to estimate the model's curvature with clarity [67]. The present study used the RMS-CCD to identify the operating variables' simple and interactive effects on the degradation of textile wastewater by the Co^2+^–BAP system. The following stages were considered for the analysis and optimization of TWW degradation: random performance of the experimental runs of the design, selecting the mathematical model (linear, quadratic, cubic) for adjustment of experimental data, analysis of variance (ANOVA), analysis of the models using 3D plots, validation of the models, and process optimization [68,69].

Design and analysis of the experiments were performed with the use of Design Expert 8.0 software (StatEase, Inc., Minneapolis, MN, USA). The effect of two independent variables (H_2_O_2_ and NaHCO_3_ concentrations) on TWW–C/F oxidation was evaluated by using a rotatable CCD. Table 3 shows the variables converted to coded values of X_1_ and X_2_ at 5 levels of –α, −1, 0, +1 and + α. Alpha (α) is the distance of each axial point (also called a star point) from the center in a central composite design. Rotatable designs provide the desirable property of constant prediction variance at all points that are equidistant from the design center, thus improving the quality of the prediction [70]. For rotatable CCD, $\alpha =\sqrt[{4}]{k}$, where k is the number of factors or independent variables studied [71]. For this research, k = 2 and the value of α = 1.1892.

**Table 3:** Levels of independent variables used in the experimental design.

Independent Variable	Range and level				
	−1.189	−1	0	+1	+1.189
**X**_**1**_: H_2_O_2_, mM	65	150	600	1050	1135
**X**_**2**_: NaHCO_3_, mM	73	90	180	270	287

It should be noted that the levels of independent variables were selected based on the results of preliminary tests. The response variables were decolorization (Y_1_), mineralization (Y_2_) and COD removal (Y_3_). The experimental design consisted of a total of 16 runs, with duplicates at the axial points and 4 replicates at the central points. The effects of each operating parameter on textile wastewater degradation and the significance of the established models were interpreted by analysis of variance (ANOVA). After the validation of the models was obtained, numerical optimization using the desirability function was performed to determine the optimal operating parameters (H_2_O_2_ and NaHCO_3_ concentrations) for maximum decolorization, mineralization, and COD removal. Under the optimum oxidation conditions, five tests were conducted to analyze the efficiency of organic matter removal using the Co^2+^–BAP system, and six physicochemical variables were characterized at the beginning and end of oxidation. Initially, an *F-*test was performed to compare variances. A two-tailed *t-*test compared the means obtained for each parameter with a confidence level of 95 % (α/2 = 0.025) to establish whether there were significant differences before and after treatment. The data were analyzed using Microsoft Excel 365 and the Real-Statistic tool.

All oxidation tests were performed in a jacketed thermostatic glass reactor at 25 ± 0.2 °C and atmospheric pressure (78 kPa) under continuous magnetic stirring at 250 rpm. For each test, the reactor was charged with 300 mL of textile wastewater previously treated by coagulation-flocculation (C/F), a specific amount of NaHCO_3_ and 45 μM Co^2+^ (Cl_2_Co.6H_2_O as the source of Co^2+^ ions). Once the pH had stabilized (approximately 10 min), H_2_O_2_ was added, and the reaction was started (t = 0). The Co^2+^ concentrations used to degrade methylene blue [42,45], reactive brilliant red X–3B [43], acid orange II [44,46], and Ponceau 4R [47] were between 5 and 20 μM. The M^2+^–BAP (M = Mn, Cu, Co) system has been shown to have excellent efficiency in decolorizing different dyes, even when small amounts of transition metals are used [35].

Textile wastewater decolorization was evaluated for 5 h, taking 0.75 mL of reactor aliquots periodically. The concentration of AB–194 as a function of time was obtained from the absorbance vs. concentration calibration curve at its maximum absorption wavelength (572 nm). The TOC concentration and COD were quantified at the beginning and end of the reaction (t = 5 h).

### 2.4 Analytical methods

The AB–194 concentration was determined from aliquots (0.75 mL of sample filtered on a 0.45 μm Millipore membrane), quantified from a previous calibration curve, and obtained by UV–Vis spectrophotometry (Genesys 150, Thermo Scientific, USA) at a wavelength (λ) of 572 nm.

Decolorization was calculated from Equation (1):

(1) $$\text{Decolorization}\;\left(\%\right)=\frac{C_{o}-C_{t}}{C_{o}}\times 100$$

where $C_{o}$ and $C_{t}$ are dye concentrations at t = 0 and at time t, respectively.

Mineralization was quantified by TOC analysis using a Multi N/C 3100 TOC analyzer (Analytik Jena, Germany) fitted with a non-dispersive infrared detector (NDIR). Mineralization or TOC removal was determined by Equation (2):

(2) $$\text{Mineralization}\left(\%\right)=\frac{{\text{TOC}}_{0}-{\text{TOC}}_{t}}{{\text{TOC}}_{0}}\times 100$$

where ${\text{TOC}}_{0}$ and ${\text{TOC}}_{t}$ are the TOC concentrations at the beginning and end of the reaction, respectively.

Chemical oxygen demand (COD) was determined by a colorimetric method (Nanocolor 500D photometer, Macherey Nagel) after digestion with K_2_Cr_2_O_7_ at 150 °C for 2 h at closed reflux. COD removal was determined by Equation (3):

(3) $$\text{COD}\;\text{removal}\left(\%\right)=\frac{{\text{COD}}_{0}-{\text{COD}}_{t}}{{\text{COD}}_{0}}\times 100$$

where ${\text{COD}}_{0}$ and ${\text{COD}}_{t}$ are the values of COD before and after oxidation, respectively.

The cobalt concentration in the aqueous solution was determined by atomic absorption spectrometry (Thermo iCE 3000 142 Series, Thermo Fisher Scientific, MA, USA) using an air/acetylene flame.

### 2.5 Chromatographic analysis of reaction byproducts

The organic compounds present in the textile wastewater (after C/F) and byproducts generated in the oxidation reaction were analyzed by GC‒MS. To separate the organic compounds, the solid phase microextraction method (SPME) was used. For this purpose, the WR/PDMS carbon fiber (10 mm, 95 μm, extraction of analytes with octanol-water partition coefficient greater than 2.0) was immersed in 5.0 mL of sample, obtained at the beginning (t = 0), at 1 h, and at the end of the reaction (t = 5 h), maintaining the stirring speed at 300 rpm for 20 min. After extraction, the fiber was retracted, and the extracted components were desorbed in a gas chromatograph coupled to a mass spectrometer (GC‒MS). The gas chromatograph was a Shimadzu GC-2010 Plus equipped with an Agilent DB-5 capillary column (30 m × 0.32 mm i.d. x 0.5 μm film thickness) coupled to a QP2010-Ultra mass spectrometer (Shimadzu, Germany) operated in selected ion monitoring mode with a heated transfer line at 290 °C. The oven temperature program was as follows: 30 °C for 2 min, from 30 to 300 °C at 10 °C/min, and 5 min hold at 300 °C. The carrier gas was helium at a 1.5 mL/min constant flow rate.

### 2.6 Ecotoxicity test

Toxicity tests with guppy fish (*Poecilia reticulata*) were carried out on treated (under optimization conditions) and textile wastewater pretreated by coagulation-flocculation (previous to oxidation). According to quality assurance and quality control procedures, bioassays assessed positive controls according to the Standard Methods for the Examination of Water and Wastewater (Section 8910C. Toxicity Test Procedures) [63] and OECD Standard Methods [72]. Young guppy fish (*Poecilia reticulata*) of both sexes 2.5 ± 0.2 cm in length were obtained from a local supplier in Manizales, Colombia. Fish were acclimatized to laboratory conditions for 10 days (T = 20 ± 2 °C, pH = 7.3 ± 0.2, dissolved oxygen 7.0 ± 1.0 mg/L, and natural photoperiod 12:12 h) in glass aquaria with dechlorinated water. The fish were fed twice daily with commercial fish feed. Unconsumed feed and animal waste were removed every two days, and water was replenished twice a week.

After this adaptation period, fish of similar average size were separated. Toxicity tests were then performed in triplicate with ten fish each, including the positive control (dechlorinated water) and TWW-C/F, before and after oxidation with the Co^2+^–BAP system under optimal degradation conditions. The water was kept semi-static (aeration with little bubbles) throughout the experiment, and the fish mortality in each group was recorded at 24 h.

### 2.7 Post-oxidation Co^2+^ adsorption

After oxidation with the Co^2+^–BAP system under optimal conditions, a batch adsorption process was carried out to remove the cobalt ions. For this purpose, 50 mg of sodium bentonite (Na–Bent) was mixed with 200 mL of the post-oxidation, and the suspension was stirred for 2 h at 25 °C and 300 rpm [62]. The adsorbent was separated by centrifugation, and the solution was filtered through a 0.45 μm membrane. Then, the cobalt concentration in the aqueous solution was measured by atomic absorption spectrometry.

## 3 Results and discussion

### 3.1 Characterization of textile wastewater

A high concentration of dyestuff, salts, biological oxygen demand (BOD_5_), chemical oxygen demand (COD), total suspended solids (TSS), variable pH, and the presence of metal ions are the main characteristics of TWW [73]. The pH range for textile sector effluents is 6.0–11.8, and the BOD_5_, COD, TSS and apparent color range from 80 to 6000 mg/L, 150 to 30,000 mg/L, 15 to 8000 mg/L, and 50 to 2500 Pt–Co units, respectively [[73], [74], [75]]. The concentration of heavy metals such as zinc, nickel, manganese, iron and copper in textile wastewater is normally less than 10 mg/L [73,74], and for chromium, the range is 2–5 mg/L [76].

The pH and concentrations of BOD_5_, COD, TSS, and total chromium for the specific case of the industrial wastewater used in this study (Table 2) are in the typical range for textile effluents. Although the apparent color in the raw textile wastewater exceeds the limit of 2500 Pt–Co units [[73], [74], [75]], after coagulation-flocculation, a greater than 98 % removal of AB‒194 dye and a decrease in apparent color of more than 94 % was achieved. Chromium in the effluent is due to the unfixed dye, since this transition metal in the metal-complex dye is part of the chromophore group [20]. Considering that dyeing with metal-complex dyes requires the use of pH regulators (sulfuric, formic, and acetic acids), electrolytes (sodium sulfate, ammonium acetate and sulfate) and leveling agents (mixtures of anionic and non-ionic surfactants) [20], conductivity, sulfate and TOC levels are very high in textile wastewater.

Table 2 shows significant differences (*p-*value <0.025) in conductivity, sulfates, COD, BOD_5_, TSS, dye concentration, total chromium, and apparent color before and after the C/F process. Parameters such as conductivity and sulfates increased with the C/F process due to adding salts to destabilize the colloids. In contrast, the other parameters decreased due to the precipitation of the colloid material. On the other hand, the *p-*value estimated for the pH and TOC measurements established non-significant differences. Although COD and BOD_5_ removals were obtained after C/F, TOC analysis was performed on the filtered samples, so the treatment did not affect this parameter.

The BOD_5_/COD ratio, or biodegradability index, is a parameter used to characterize the biodegradability of an effluent. Accordingly, wastewater with a BOD_5_/COD ratio ≥0.4 corresponds to effluents readily treatable by biological treatments [77,78]. The textile wastewater used in this study has an index of 0.29 ± 0.01 after the coagulation/flocculation process, indicating that it is not biodegradable.

### 3.2 Experimental design - oxidation tests

Experimental values of the runs carried out in the experimental design and responses observed for 16 experiments are shown in Table 4.

**Table 4:** Codified and experimental values of runs performed and results obtained in the oxidation of textile wastewater.

Run	Std	Values				Response variables (%)		
		Codified		Experimental (mM)		Decolorization	TOC	COD
		X_1_	X_2_	X_1_	X_2_	Y_1_	Y_2_	Y_3_
1	2	+α	0	1135	180	89.11	22.13	44.83
2	14	0	0	600	180	93.67	28.08	47.55
3	13	0	0	600	180	93.62	28.17	46.23
4	8	+1	−1	1050	90	72.98	15.41	39.77
5	15	+α	0	1135	180	89.68	24.28	45.67
6	10	0	0	600	180	95.9	29.47	46.4
7	3	+1	+1	1050	270	74.5	18.25	40.06
8	4	0	-α	600	73	51.32	10.67	31.47
9	6	0	-α	600	73	55.68	11.4	26.62
10	9	-α	0	65	180	29.98	7.69	22.5
11	7	−1	+1	150	270	31.71	1.1	25.5
12	5	0	+α	600	287	64.82	12.47	33.79
13	11	0	0	600	180	91.28	27.49	45.5
14	1	0	+α	600	287	63.06	11.47	33.79
15	12	-α	0	65	180	29.99	7.68	22.5
16	16	−1	−1	150	90	18.36	1.65	8.46

A multiple regression analysis was conducted, and response functions were fitted to a second-order polynomial equation to determine the coefficients of the response models and the significance of these coefficients. The model's significance was estimated using analysis of variance (ANOVA). In Table 5, Table 6, Table 7, the results for the three response functions are presented. The *F-*value of a model or its term should be distant from one, as a value close to one indicates a similar effect for each term on the response, and the *p-*value should be as low as possible, at least lower than 0.05 [79,80]. For this study, all three models showed *p*-values <0.0001, which indicates that they are highly significant and can be used to predict response functions. A model with a 95 % confidence level is considered significant if the *p-*value <0.05. The quality of the model's fits was evaluated by the coefficients of determination (R^2^, Adjusted-R^2^, Predicted-R^2^), adequate precision, and coefficient of variation (CV).

**Table 5:** ANOVA results for decolorization (%) - Model and coefficients validation.

Source	Sum of squares	Degrees freedom	Mean square	*F-*value	*p*-value
Model regression	10582.21	5	2116.44	352.45	<0.0001^a^
X_1_	5900.81	1	5900.81	982.64	<0.0001^a^
X_2_	163.22	1	163.22	27.18	0.0004^a^
X_1_X_2_	34.99	1	34.99	5.83	0.0364^a^
X_1_^2^	2817.74	1	2817.74	469.23	<0.0001^a^
X_2_^2^	2992.87	1	2992.87	498.39	<0.0001^a^
Residual	60.05	10	6.01		
Lack of fit	38.16	3	12.72	4.07	0.0576^b^
Pure error	21.89	7	3.13		
Cor total					

R^2^ = 0.9944, Adj-R^2^ = 0.9915, Pred-R^2^ = 0.9816, CV = 7.4568 %, Adeq-Precision = 36.8625.

a Significant at 95 % confidence interval.

b Non-significant at 95 % confidence interval.

**Table 6:** ANOVA results for mineralization (%) - Model and coefficients validation.

Source	Sum of squares	Degrees freedom	Mean square	*F-*value	*p*-value
Model regression	1353.29	5	270.66	188.06	<0.0001^a^
X_1_	476.34	1	476.34	330.98	<0.0001^a^
X_2_	2.11	1	2.11	1.47	0.2538^b^
X_1_X_2_	2.87	1	2.87	2	0.188^b^
X_1_^2^	401.58	1	401.58	279.03	<0.0001^a^
X_2_^2^	710.36	1	710.36	493.58	<0.0001^a^
Residual	14.39	10	1.44		
Lack of fit	9.22	3	3.07	4.16	0.0548^b^
Pure error	5.17	7	0.74		
Cor total					

R^2^ = 0.9895, Adj-R^2^ = 0.9842, Pred-R^2^ = 0.9643, CV = 3.7496 %, Adeq-Precision = 51.4543.

a Significant at 95 % confidence interval.

b Non-significant at 95 % confidence interval.

**Table 7:** ANOVA results for COD removal (%) - Model and coefficients validation.

Source	Sum of squares	Degrees freedom	Mean square	*F-*value	*p*-value
Model regression	1935.64	5	387.13	97.45	<0.0001^a^
X_1_	1035.1	1	1035.1	260.55	<0.0001^a^
X_2_	84.79	1	84.79	21.34	0.001^a^
X_1_X_2_	70.14	1	70.14	17.66	0.0018^a^
X_1_^2^	389.83	1	389.83	98.12	<0.0001^a^
X_2_^2^	569.58	1	569.58	143.37	<0.0001^a^
Residual	39.73	10	3.97	0	
Lack of fit	25.45	3	8.48	4.16	0.0549^b^
Pure error	14.27	7	2.04		
Cor total					

R^2^ = 0.9799, Adj-R^2^ = 0.9698, Pred-R^2^ = 0.9277, CV = 5.6883 %, Adeq-Precision = 29.8434.

a Significant at 95 % confidence interval.

b Non-significant at 95 % confidence interval.

The high values of R^2^ and adjusted-R^2^ for the three response variables, which were higher than 0.9799 and 0.9698, respectively, confirm the validity of the proposed models [78,81]. The predicted-R^2^ values reasonably agree with the adjusted-R^2^ when the difference between them is less than 0.2 [78,82]. This study's differences were 0.0099, 0.0199 and 0.0421 for decolorization, mineralization and COD removal, respectively. Adequate precision measures the signal-to-noise ratio and compares the range of the predicted values at the design points to the average prediction error [83].

In this research, the values of adequate precision were between 29.84 and 51.45, and values > 4 are desirable for models. It is considered good for a model's coefficient of variance (CV) to be less than 10 % [78,84]. The CV values of the three response variables were between 3.75 and 7.46 %, indicating low dispersion between the fitted and actual data.

Analysis of variance (ANOVA) is usually accompanied by a model validation statistic called a lack of fit test. The lack of fit values were not significant for the decolorization, mineralization, and TOC removal models, indicating that the regression models adequately described the relationships between the experimental factors and the response function. Consistently, a non-significant lack of fit values is desirable [85]. The linear coefficients for H_2_O_2_ in the three response functions were the most significant, followed by the quadratic and interaction coefficients. The ANOVA study suggested that H_2_O_2_ concentration (*p-*value <0.0001, and sum square of 5900, 476, and 1035 for the decolorization, mineralization, and COD removal models) significantly affected textile wastewater degradation.

The response functions determined for decolorization (Y_1_), mineralization (Y_2_) and COD removal (Y_3_) are presented in Equations (4), (5), (6), respectively:

(4) $$Y_{1}=-88.28247+0.20065X_{1}+1.11409X_{2}-7.30247\times 10^{-5}X_{1}X_{2}-1.10481\times 10^{-4}{X_{1}}^{2}-2.84610\times 10^{-3}{X_{2}}^{2}$$

(5) $$Y_{2}=-40.01430+0.06189X_{1}+0.49181X_{2}+2.09259\times 10^{-5}X_{1}X_{2}-4.17084\times 10^{-5}{X_{1}}^{2}-1.38658\times 10^{-3}{X_{2}}^{2}$$

(6) $$Y_{3}=-39.78556+0.090931X_{1}+0.54194X_{2}-1.03395\times 10^{-4}X_{1}X_{2}-4.10936\times 10^{-5}{X_{1}}^{2}-1.24161\times 10^{-3}{X_{2}}^{2}$$

where X_1_ and X_2_ represent the concentrations of H_2_O_2_ (mM) and NaHCO_3_ (mM), respectively.

According to the coefficients of Equations (4), (5), (6), decolorization, mineralization, and COD removal were improved with increasing H_2_O_2_ and NaHCO_3_ concentrations (coefficients with positive signs). The interactive effects of H_2_O_2_–NaHCO_3_ ($X_{1}X_{2}$) were significant in the response functions decolorization and COD removal (*p-*value <0.05) and non-significant for mineralization. Although NaHCO_3_ is an activator of H_2_O_2_, high concentrations of sodium bicarbonate do not favor textile wastewater degradation.

**Fig. 1:**
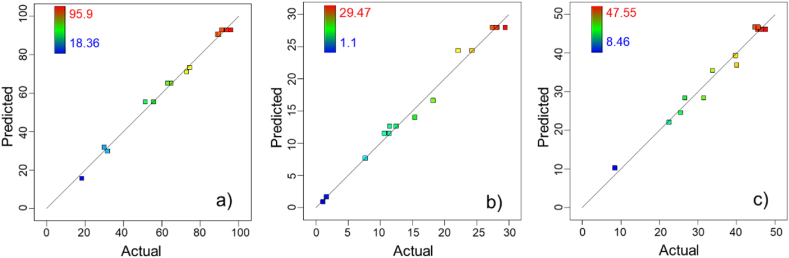
Plots of predicted vs. actual values for textile wastewater degradation. a) Decolorization, b) mineralization, and c) COD removal.

**Fig. 2:**
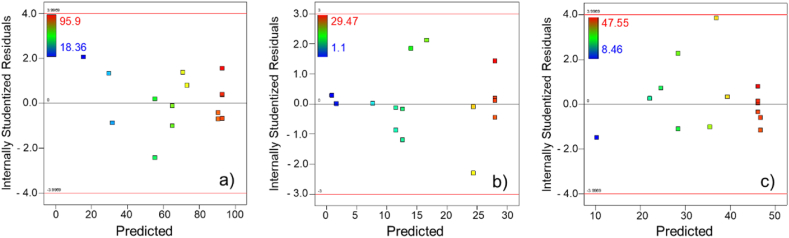
Plots of studentized residuals vs. predicted for textile wastewater degradation. a) Decolorization, b) mineralization, and c) COD removal.

Verifying the proposed models is fundamental to the analysis procedure and ensures that the models provide a good approximation of the system under study. Diagnostic plots of “Actual vs. Predicted” (Fig. 1) and “Residual vs. Predicted” (Fig. 2) values were used to estimate the adequacy of the regression models.

In Fig. 1, the data were well distributed around the straight-line X = Y in a narrow zone, indicating that the proposed models for decolorization (Fig. 1a), mineralization (Fig. 1b), and COD removal (Fig. 1c) had an adequate approximation to the true value. The “Residual vs. Prediction” plots that evaluated decolorization (Fig. 2a), mineralization (Fig. 2b), and COD removal (Fig. 2c) showed random behavior and the formation of a horizontal band around the residual line equal to zero. These plots are also used to examine outliers, and for the present study, there were no residuals outside the red lines on the graphs.

**Fig. 3:**
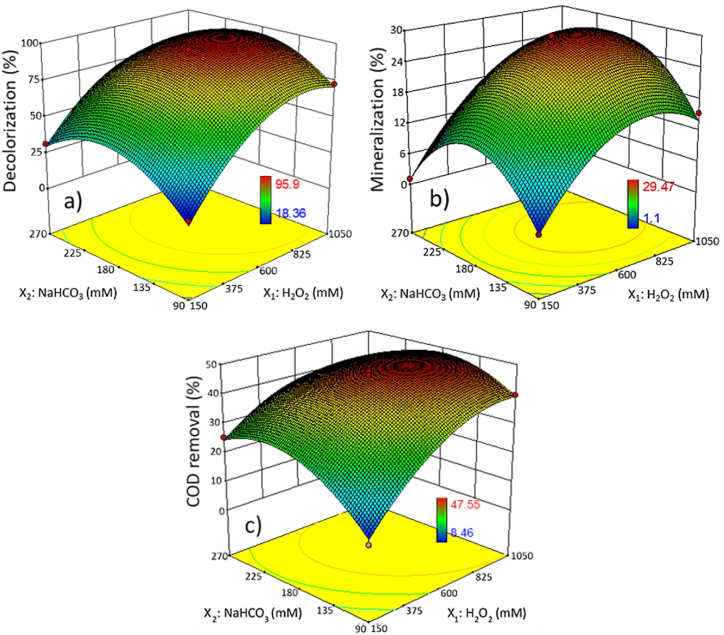
3D response surface plots for textile wastewater degradation as a function of H_2_O_2_ and NaHCO_3_ concentrations. a) Decolorization, b) mineralization, and c) COD removal.

Fig. 3 shows the 3D response surface plots for the combined effect of H_2_O_2_ and NaHCO_3_ concentrations on TWW–C/F degradation at a Co^2+^ concentration of 45 μM and reaction time of 5 h. The highest decolorization (Fig. 3a), mineralization (Fig. 3b), and COD removal (Fig. 3c) were obtained at medium-high H_2_O_2_ concentrations (670–1040 mM) and medium NaHCO_3_ concentrations (150–210 mM). In the oxidation of the reactive brilliant red X–3B (67.5 μmol/L) by the Co^2+^–BAP system, it was found that an increase in NaHCO_3_ concentration from 10 to 50 mM decreases the degradation efficiency. The degradation efficiency of X–3B dye was attributed to the formation of the Co^2+^–HCO_3_^–^ complex, which produces more active oxygen radicals in the system. However, at higher NaHCO_3_ concentrations, the reaction rate decreased due to the dissociation of HCO_3_^−^ to CO_3_^2−^, which can form more stable complexes with Co^2+^ ions [43]. In addition, free HCO_3_^−^ in solution at higher concentrations may act as a scavenger of hydroxyl radicals (•OH) [35,43]. When the H_2_O_2_ concentration increases above an optimum value, H_2_O_2_ can also act as a scavenger of •OH, with the subsequent formation of perhydroxyl radicals (${\text{HO}}_{2}^{•-}$), which have a considerably lower oxidation potential [43,86].

Color removal in textile wastewater does not necessarily suggest complete degradation under CO_2_ and H_2_O. Specific organic compounds in the initial sample or the reaction intermediates formed during the degradation of dyes are colorless [48]. After 5 h of reaction, this study obtained important decolorization (18.4–95.9 %) and incomplete mineralization (1.1–29.5 %) of TWW–C/F. The differences between decolorization and mineralization measure colorless organic compounds that contribute to TOC but not color measurements.

The proposed reaction mechanism in the Co^2+^–BAP system assumes the in-situ formation of complexes between Co^2+^ ions and HCO_3_^−^ ions, and the intermediate complexes act as catalysts in activating H_2_O_2_ to produce ROS [35,42]. By cyclovoltammetry measurements, the in-situ formation of the [Co^2+^(HCO_3_^−^)]^+^ complex at HCO_3_^−^ concentrations of 5–10 mmol/L and the existence of another complex [Co^2+^(HCO_3_^−^)^2^] at high concentrations (50–100 mmol/L HCO_3_^−^) were suggested [42]. In addition, the stability of the Co^2+^–HCO_3_^−^ complex could be enhanced with the presence of organic dye as a coordinating ligand [42,87]. From the results of photoluminescence probing technology, electron spin resonance, and radical trap experiments [46], it was deduced that the •OH radicals produced by the system were not free but were closely associated with the cobalt complex. However, a detailed mechanism for the Co^2+^–HCO_3_^–^ system, especially the generation and action of the “crypto-•OH” radical, is still lacking [44].

Studies on BAP technology show that HCO_3_^−^ has the particularity of activating H_2_O_2_ to form peroxymonocarbonate ions ($\text{HC}O_{4}^{-}$) according to the following reaction [35,36]:

(7) $$HCO_{3}^{-}+H_{2}O_{2}↔H_{2}O+HCO_{4}^{-}$$

Chemiluminescence studies showed that $HCO_{4}^{-}$ in the BAP system can decompose into other reactive species, such as ^1^ $O_{2}$, $O_{2}^{•-}$, and ${CO}_{3}^{•-}$ [36,[88], [89], [90]] which are generated in the following reactions:

(8) $$\text{HC}O_{4}^{-}\to {\text{CO}}_{3}^{•-}+•\text{OH}$$

(9) $${\text{CO}}_{3}^{•-}+H_{2}O_{2}\to \text{HC}O_{3}^{-}+{\text{HO}}_{2}^{•}$$

(10) $${\text{HO}}_{2}^{•}\to H^{+}+O_{2}^{•-}$$

(11) $$•\text{OH}+{\text{HO}}_{2}^{•}\to H_{2}O+O_{2}^{•-}$$

In addition, the carbonate radical anion can be produced by the scavenging of •OH, as shown in the following equation [91]:

(12) $$•\text{OH}+\text{HC}O_{3}^{-}\to H_{2}O+{\text{CO}}_{3}^{•-}$$

Based on the experimental results obtained for the oxidative degradation of methylene blue (MB) and acid orange II (AOII) with the Co^2+^–BAP system, Xu et al. (2011) [42] and Guo et al. (2015) [92] proposed the following reaction mechanism:

(13) $${\text{Co}}^{\text{II}}+\text{HC}O_{3}^{-}↔{\left[{\text{Co}}^{\text{II}}(\text{HC}O_{3}^{-}\right]}^{+}$$

(14) $${\left[{\text{Co}}^{\text{II}}(\text{HC}O_{3}^{-}\right]}^{+}+\text{Dye}↔{\left[{\text{Co}}^{\text{II}}\left(\text{HC}O_{3}^{-}\right)\left(\text{Dye}\right)\right]}^{+}$$

(15) $${\left[{\text{Co}}^{\text{II}}\left(\text{HC}O_{3}^{-}\right)\left(\text{Dye}\right)\right]}^{+}+H_{2}O_{2}↔{\left[{\text{Co}}^{\text{II}}\left(\text{HC}O_{3}^{-}\right)\left(\text{Dye}\right)\left(H_{2}O_{2}\right)\right]}^{+}$$

(16) $${\left[{\text{Co}}^{\text{II}}\left(\text{HC}O_{3}^{-}\right)\left(\text{Dye}\right)\left(H_{2}O_{2}\right)\right]}^{+}\to {\left[{\text{Co}}^{\text{III}}\left(\text{HC}O_{3}^{-}\right)\left(\text{Dye}\right)\left(•\text{OH}\right)\right]}^{2+}+{\text{OH}}^{-}$$

(17) $${\left[{\text{Co}}^{\text{III}}\left(\text{HC}O_{3}^{-}\right)\left(\text{Dye}\right)\left(•\text{OH}\right)\right]}^{2+}\to {\left[{\text{Co}}^{\text{III}}\left(\text{HC}O_{3}^{-}\right)\right]}^{2+}+\text{Oxidation}\;\text{products}$$

(18) $${\left[{\text{Co}}^{\text{III}}\left(\text{HC}O_{3}^{-}\right)\right]}^{2+}+H_{2}O_{2}\to {\left[{\text{Co}}^{\text{II}}\left(\text{HC}O_{3}^{-}\right)\right]}^{+}+{\text{HO}}_{2}^{•}+H^{+}$$

### 3.3 Optimization and model validation

**Fig. 4:**
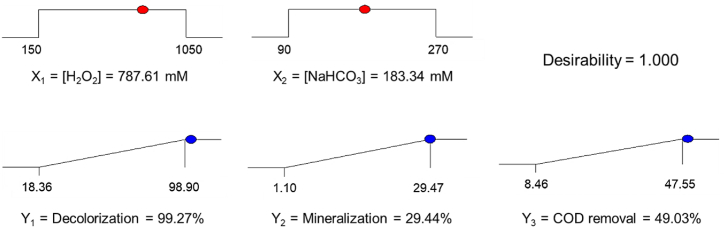
Desirability ramp for multi-objective optimization.

The optimum reagent concentrations that maximize the degradation of TWW–C/F were determined using the desirability function of the Design Expert software, and the results are presented in Fig. 4. The desirability function is the most popular tool to optimize multiple responses [93,94]. Desirability always takes values between 0 and 1, with zero (0) indicating an undesirable response and one (1) indicating an utterly desirable value, i.e., an ideal response. Intermediate values of desirability indicate more or less desirable responses [94]. Oxidation conditions for degradation of textile wastewater with the Co^2+^–BAP system guaranteeing maximum decolorization, mineralization, and COD removal are 787.61 mM H_2_O_2_ and 183.34 mM NaHCO_3_, maintaining the Co^2+^ concentration at 45 μM.

Additional tests were carried out to check the validity of the predicted models under the obtained multi-objective optimization conditions with concentrations not evaluated in the CCD, and the results are summarized in Table 8. The errors between the predicted and experimental values for multi-objective optimization were less than 3.0 for decolorization and mineralization and close to 7.0 % for COD removal. Therefore, the models generated by RSM were accurate enough to predict the degradation of textile wastewater by the Co^2+^–BAP system.

**Table 8:** Comparison of predicted and experimental results for validation points of the experimental design.

Test	H_2_O_2_ (mM)	NaHCO_3_ (mM)	Response functions (%)					
	X_1_	X_2_	Y_1_		Y_2_		Y_3_	
			Pred.	Exp.	Pred.	Exp.	Pred.	Exp.
Optimal multi-objective	787.61	183.34	99.27	>99.40	29.44	30.20	49.03	52.02
Validation 1	900.00	120.00	87.64	84.27	23.21	24.71	44.75	47.80
Validation 2	450.00	150.00	77.79	79.85	23.38	23.96	39.18	42.10

Table 9 summarizes the physicochemical characterization of the TWW–C/F before and after oxidation with the Co^2+^–BAP system under optimal degradation conditions and *p-*values obtained from the statistical analysis. The *p-*values for COD, BOD_5_, TOC, dye concentration, and apparent color are less than 0.025, indicating significant differences before and after oxidation treatment. However, the pH obtained a *p-*value of 0.26, which suggests that there were non-significant changes in this variable since the NaHCO_3_ in the reaction medium forms a buffer solution.

**Table 9:** Physicochemical characterization of textile wastewater before and after oxidation with the Co^2+^–BAP system.

Parameter	Before oxidation	After oxidation Optimal conditions	*p*-value
pH	8.9 ± 0.3	8.7 ± 0.2	0.26^a^
COD (mg O_2_/L)	1428 ± 84	685 ± 40	3.13 × 10^−7^^b^
BOD_5_ (mg O_2_/L)	419 ± 32	307 ± 13	7.21 × 10^−5^^b^
BOD_5_/COD	0.29 ± 0.01	0.45 ± 0.01	–
TOC, mg C/L	1083 ± 68	756 ± 37	1.41 × 10^−5^^b^
COD/TOC	1.32 ± 0.01	0.91 ± 0.01	–
Acid Black 194, mg/L	16.6 ± 1.2	≤0.09	3.00 × 10^−5^^b^
Apparent color, U. Pt–Co	2545 ± 21	184 ± 3	3.45 × 10^−8^^b^

a Non-significant difference.

b Significant difference.

Under optimal degradation conditions, complete decolorization (≥99.40) and total removal of AB–194 (≥99.40 %) were obtained. In the oxidation of acid orange II (125 μM) by the Co^2+^–HCO_3_^-^ system and H_2_O_2_ (50 mM), TOC and COD removals of 45.3 and 78.7 % were achieved, respectively; values higher than those obtained in this study, 49.03 % COD removal and 29.44 % mineralization. However, it is not possible to compare the TOC and COD removals in industrial wastewater and simulated with a model molecule. Reactive Black 5 (RB5) diazodye and industrial wastewater with this same commercial dye (Setazol Black DPT) were treated by ozone-based AOPs and H_2_O_2_/UV processes. For industrial wastewater, the COD and TOC decreases were low, 10 % of COD and 20 % of TOC, in contrast to the simulated values of 90 % and 50 %, respectively [30].

The COD/TOC ratio variation is related to changes in the chemical properties of pollutants in the wastewater after a specific treatment. In some cases, the value of this ratio determines the amount of oxygen needed to oxidize organic matter relative to the carbon content in the structure of its molecules [95]. The COD/TOC ratio before and after the oxidation of the textile wastewater by Co^2+^–BAP had a reduction of 31 %, which indicates a transformation of organic compounds into intermediates.

### 3.4 Chromatographic analysis

**Fig. 5:**
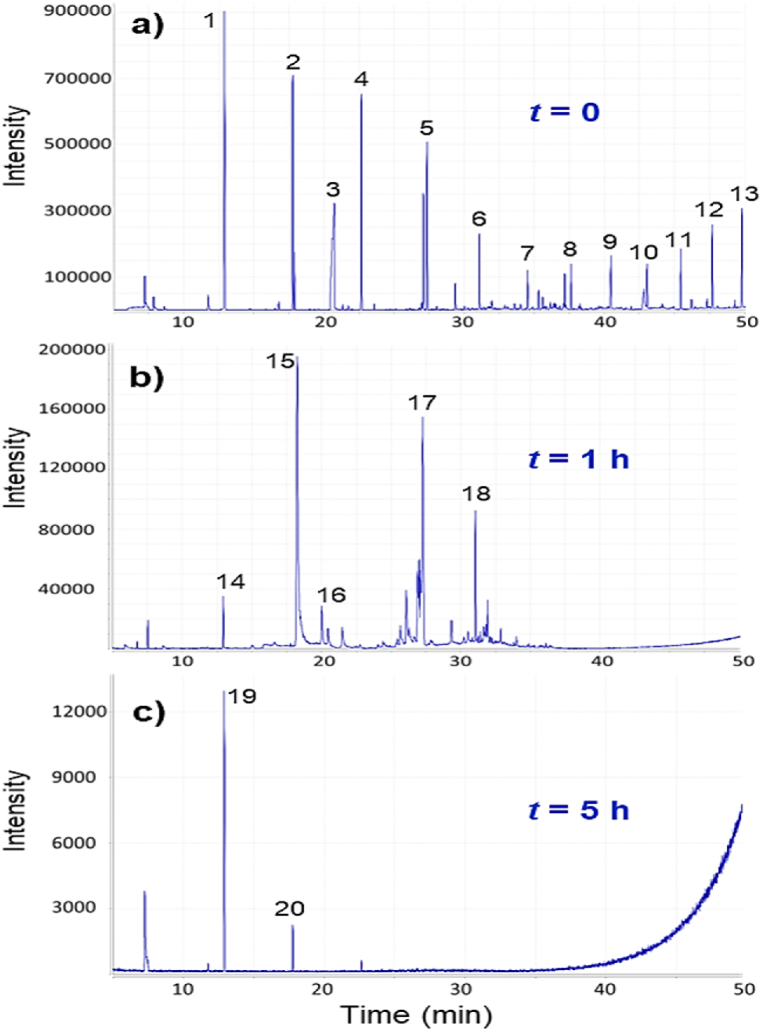
GC-MS chromatograms after SPME extraction from a) TWW–C/F before oxidation, b) at t = 1 h of oxidation and c) TWW–C/F treated by Co^2+^–BAP.

Fig. 5 shows the GC‒MS chromatograms obtained after the SPME extraction process at the beginning (t = 0), at reaction time t = 1 h, and at the end of the reaction (t = 5 h). Preliminary identification of the compounds was performed from their mass fragments (*m*/*z*) compared with the NIST 2010 library (Table 10). More than 13 signals were observed in the textile wastewater before oxidation (Fig. 5a), and the identified compounds are characterized by high molar mass and correspond mostly to silicon-based softening agents. Silanes and multifunctional silicones are the most widely used group of softeners in the last two decades and react with the fiber through cross-linking and strong covalent bonds, creating prolonged effects such as color performance and wrinkle recovery [96,97]. These finishing agents provide softness, improved feel, drape, and durability in the wash and are applied to the fiber in the last stage of the dyeing process [98]. After t = 1 h of oxidation (Fig. 5b), a decrease in the number and intensity of signals is observed, and at the end of the oxidation with the Co^2+^–BAP system (Fig. 5c), practically all the compounds have been degraded. The degradation of organic compounds after oxidation of TWW–C/F with the Co^2+^–BAP system is consistent with the increase in the biodegradability index (BOD_5_/COD ratio), which went from 0.29 ± 0.01 in the untreated water to 0.45 ± 0.01 in the treated water.

**Table 10:** Summary of mass spectra of major organic compounds recovered from textile wastewater.

Peak	Retention time (min)	Molecular ion (*m*/*z*)	Formula	Principal fragments *m*/*z*
1, 14, 19	12.892	296	C_8_H_24_O_4_Si_4_	73, 281
2, 15, 20	17.771	370	C_10_H_30_O_5_Si_5_	73, 267, 355
3	20.733	113	C_6_H_11_NO	55, 41, 85
4	22.668	444	C_12_H_36_O_6_Si_6_	73, 147, 341, 429
5, 17	27.101	518	C_14_H_42_O_7_Si_7_	73, 147, 281, 327, 415
6, 18	31.080	458	C_14_H_42_O_5_Si_6_	73, 147, 221, 281, 355
7	34.535	504	C_14_H_44_O_6_Si_9_	73, 147, 207, 355, 489
8	37.627	576	C_18_H_52_O_7_Si_7_	73, 147, 221, 281, 415
9	40.466	532	C_16_H_48_O_6_Si_7_	73, 147, 221, 355, 429
10	43.057	592	C_16_H_48_O_8_Si_8_	73, 147, 221, 355, 489
11	45.453	666	C_18_H_54_O_9_Si_9_	73,147, 221, 429, 651
12	47.704	740	C_20_H_54_O_9_Si_9_	73, 147, 221, 429, 709
13	49.820	888	C_18_H_60_O_10_Si_10_	147, 221, 355, 429, 651
16	20.025	178	C_8_H_18_O_4_	43, 69, 95, 111

### 3.5 Toxicity test on guppy fish (*Poecilia reticulata*)

No mortality or altered behavior occurred during the acclimatization of the fish. The results of the toxicity tests are summarized in Table 11. For TWW–C/F corresponding to the sample before oxidation, during the second hour of the bioassay, fish showed momentary paralysis of the fins, and between 3 and 6 h, all fish died. Between 20 and 30 % of the dead fish showed curvature of the spinal column, which may be associated with nervous system involvement or failure [99].

**Table 11:** Data on survival of *Poecilia reticulata* exposed to the control solution (dechlorinated water) and TWW–C/F before and after oxidation with the Co^2+^–BAP system.

	Number fish	Time						Survival (%)
		4 h	8 h	12 h	16 h	20 h	24 h	
Control (dechlorinated water)	10	10	10	10	10	10	10	100
	10	10	10	10	10	10	10	
	10	10	10	10	10	10	10	
TWW–C/F Before Oxidation	10	2	0	0	0	0	0	0
	10	2	0	0	0	0	0	
	10	2	0	0	0	0	0	
TWW–C/F After oxidation	10	10	10	10	10	10	10	100
	10	10	10	10	10	10	10	
	10	10	10	10	10	10	10	

In the biotests after oxidation of the TWW–C/F with the Co^2+^–BAP system, there were no fish kills, and compared to the untreated wastewater (TWW–C/F), a total reduction in acute toxicity was achieved. The fish showed normal behavior during the first 3 h of the post-oxidation test. However, between 4 and 6 h, the fish showed accelerated movements and momentary startle reactions, returning to their natural behavior. Finally, the surviving fish from the tests were brought to a tank with aerated water for recovery, and after 48 h, they were all alive.

### 3.6 Post-oxidation Co^2+^ adsorption

Cobalt ions (Co^2+^) used as a catalyst in the BAP system must be removed after textile wastewater oxidation since these metal ions are toxic to aquatic ecosystems [100]. Under the multi-objective optimization conditions, the Co^2+^ concentration in the reaction medium was 45 μM, equivalent to 2.65 mg/L. The initial concentration of the metal ion in the aqueous phase provides the driving force to overcome the resistance to metal mass transfer from the aqueous solution to the solid phase of the adsorbent. Given the low concentration of Co^2+^ in the reaction medium, three successive adsorptions were necessary to eliminate cobalt ions to a value below the discharge limit according to Colombian legislation, which is 0.5 mg/L.

**Fig. 6:**
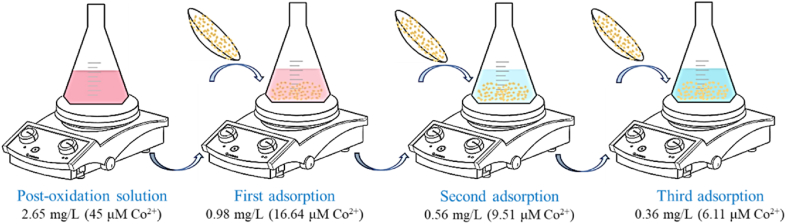
Scheme for consecutive adsorption for the removal of Co^2+^ ions. Adsorption conditions: Post-oxidation solution volume = 200 mL, adsorbent mass = 50 mg of Na–Bent, sped stirring = 300 rpm, pH = 8.5, and contact time = 120 min.

The scheme of the post-oxidation Co^2+^ adsorption is shown in Fig. 6. The cobalt removals in the first, second, and third adsorption were 63.0, 42.9 and 35.7 %, respectively. As the Co^2+^ concentration decreased, removing the ions was more difficult since the concentration gradient's driving force was smaller.

## 4 Conclusions

In this study, the Co^2+^–BAP system was successfully applied to degrade textile wastewater contaminated with the azo dye Acid Black 194. This wastewater is a complex matrix due to the high concentrations of TOC and COD associated with the metal-complex dye and additives (such as softening agents) used in the dyeing process.

Using RSM based on a CCD, the optimal reagent concentrations (787.61 mM H_2_O_2_ and 183.34 mM NaHCO_3_) that maximize the degradation of TWW–C/F (decolorization ≥99.40 %, 32.20 % mineralization, and 52.02 % chemical oxygen demand removal) were obtained. Although the degree of mineralization obtained with the Co^2+^–BAP system was low, this AOP improved the biodegradability of TWW–C/F and significantly reduced toxicity, making it possible to post-treat textile wastewater under a biological process.

The cobalt (catalyst) in the post-oxidation solution is a toxic water pollutant, so it was absorbed with sodium bentonite. Three consecutive adsorptions with Na–Bent removed 86.4 % of the cobalt ions.

## Data availability statement

Data used and/or analyzed during the current study are available from the corresponding author upon reasonable request.

## CRediT authorship contribution statement

**Francisco J. Ariza-Pineda:** Investigation, Methodology, Writing – original draft, Formal analysis. **Iván F. Macías-Quiroga:** Investigation, Methodology, Writing – original draft, Writing – review & editing, Formal analysis, Conceptualization. **Diego F. Hinojosa-Zambrano:** Investigation, Methodology. **Juan D. Rivera-Giraldo:** Investigation, Methodology, Formal analysis. **Diana M. Ocampo-Serna:** Formal analysis, Investigation, Methodology. **Nancy R. Sanabria-González:** Conceptualization, Formal analysis, Investigation, Methodology, Writing – original draft, Writing – review & editing.

## Declaration of competing interest

The authors declare that they have no known competing financial interests or personal relationships that could have appeared to influence the work reported in this paper.
